# Hematuria as the first sign of multiple myeloma

**DOI:** 10.1002/ccr3.1062

**Published:** 2017-07-10

**Authors:** Mariana Alves, Raul Moreno, Fátima Rodrigues, Anabela Rodrigues, Teresa Fonseca

**Affiliations:** ^1^ CHLN Medicina III Hospital Pulido Valente Lisboa Portugal; ^2^ CHLN Hematologia e Transplantação Medular Hospital Santa Maria Lisboa Portugal; ^3^ CHLN Imuno‐hemoterapia Hospital Santa Maria Lisboa Portugal

**Keywords:** hematuria, lupus coagulation inhibitor, multiple myeloma

## Abstract

Patients with multiple myeloma may present with several signs and symptoms, inclusive of hemorrhagic diathesis. This case emphasizes the need to suspect uncommon etiologies for common signs and to be aware of the atypical effects of paraproteinemia.

## Background

Myeloma multiple (MM) is a clonal plasma cell malignant neoplasm that accounts for approximately 10% of hematologic malignant disorders. It has an incidence of 4 of 100,000 habitants/year in the Western World 1, 2, 3.

The most common presenting symptoms of MM are fatigue and bone pain 1. Although patients with high titer of serum paraprotein can manifest hemostatic abnormalities, which could be a diagnostic challenge 3, 4, 5, most are predisposed to hemorrhage, which can range from skin hemorrhages to life‐threatening gastrointestinal bleeding. MM is associated with a bleeding diathesis in about 15% of patients 4. Less commonly, plasma cell disorders can occur with thrombotic complications, especially in the presence of lupus anticoagulant (LA) as a paraneoplastic syndrome 4.

There are several possible etiologies to hemorrhagic diathesis: platelet dysfunction (the most common), autoimmune thrombocytopenia, inhibition of polymerization of fibrin monomers, monoclonal thrombin inhibitor, circulating heparin‐like anticoagulant, coagulation factors deficiency, acquired von Willebrand syndrome, local‐issue fragility secondary to amyloidosis, and vascular endothelium damage 3, 4. Regardless of the mechanism of the bleeding event, the mainstay therapy is treatment for the underlying disease 3, 4.

## Case Report

A leukodermic 60‐year‐old woman with a prior history of dyslipidemia and hypertension was admitted to the emergency department due to the presence of gross hematuria over 15 days. It had been previously empirically treated as a urinary infection without improvement. Patient denied previous history of hemorrhagic dyscrasias, such as ecchymosis, epistaxis, gingival bleeding or hemarthrosis even after two surgeries (cesarean, appendicectomy), and several dental extractions. The hematology values at the emergency room were as follows: leukocytes, 7.24 × 10 9/L; hemoglobin, 9.0 g/dL; mean corpuscular volume, 100 fL; platelets, 134 × 10 9/L; activated partial thromboplastin time (aPTT), > 100 sec; and prothrombin time (PT) ratio, 1.14. Urinalysis confirmed that hematuria and urine culture was negative. As bleeding did not produce hemodynamic instability, after urologic evaluation, the patient was referred to an internal medicine appointment.

Ultrasound imaging of the urinary system found signs of kidney microlithiasis without any other abnormality.

Extended blood tests revealed persistent anemia (hg 8.0 g/dL) and macrocytosis (MCV 100 fL), normal B12 vitamin and folic acid, normal iron deposits (ferritin 50.4 ng/mL), and reticulocytosis (3.3%). Abnormal coagulation parameters persisted. The aPTT was not corrected after incubation with mixture test, and determination of coagulation factors detected low values of FVIII, FIX, FXI, and FXII; normal values of FV; and high values of FVII, von Willebrand factor (vWF):Ag, and vWF:f (Table 1). Blood type was O Rh+.

**Table 1:** Blood tests results

	Reference values	Initial	Postchemotherapy	Post‐transplant
Hemoglobin (g/dL)	12.0–15.3	9.0	11.5	11.5
Mean corpuscular volume (fL)	80.0–97.0	100	94.2	92.9
Leukocytes (×10^9^/L)	4.0–11.0	7.24	4.45	5.56
Platelets (×10^9^/L)	150–450	134	143	101
Reticulocytes (%)		3.3	ND	2.2
Activated partial thromboplastin time (sec)	31	>100.0	73.8	36.6
Prothrombin time (PT) (sec)	11.6	13.3	11.9	11.1
PT ratio (INR)		1.14	1.02	0.96
D‐dimers (*μ*g/mL)	0.0–0.25	<0.15	<0.15	<0.15
Urea (mg/dL)	10–50	29	25	39
Creatinine (mg/dL)	0.5–1.1	0.9	0.75	0.75
Calcemia (mg/dL)	8.6–10.2	10.0	10.2	9.6
Lactate dehydrogenase (U/L)	208–378	195	179	154
Albumin (g/dL)	3.2–4.8	3.6	4.4	4.6
AST/ALT (U/L)	10–49	13/17	20/23	20/21
GGT (U/L)	<38	13	19	32
Total bilirubin (mg/dL)	<1.0	0.14	0.17	0.13
Iron (ug/dL)	50–170	107.9	144	ND
Ferritin (ng/mL)	10–291	50.4	1039	ND
B12 vitamin (pg/mL)	210–910	537	ND	ND
Folate (ng/mL)	>5.4	9.8	ND	ND
Factor V (%)	50–100	84	ND	ND
Factor VII (%)	70–130	118	120	165
Factor VIII (%)	50–150	45	89	174
Factor IX (%)	50–130	28	88	190
Factor XI (%)	50–130	21	79	173
Factor XII (%)	70–130	11	70	247
vWF: func (%)	40,3–125,9 (ORh+)	375	219	211
vWF: Ag	42,0–140,6 (ORh+)	461	240	210
Fibrinogen (mg/dL)	200–400	242	392	257
Beta‐2 microglobulin (mg/L)	1.0–2.4	4.10	3.70	2.77
IgG/IgA/IgM (mg/dL)	751–1560/82–453/46–304	3420/11/16	1020/43/464	500/<5.0/20
Kappa/lambda (mg/dL)	629–1350/313–723	2800/32 (K/L:87,5)	464/94 (K/L:4,96)	123/52 (K/L:2,37)
Col/epinephrin (seg)	71–118	>300	>300	ND
Col/ADP (seg)	85–165	>300	260	ND
Lupus anticoagulant	Negative	Positive	Positive	Negative

ND = Not determined; Func = functional; AG = Antigen; vWF = von Willebrand factor.

Determination of LA was positive, while anti‐cardiolipin and anti‐Beta‐2 glycoprotein antibodies were negative. PFA‐100 presented prolongation of both test results (Col/Epinephrin and Col/ADP > 300). Screening for autoimmune disease presented negative results.

**Figure 1:**
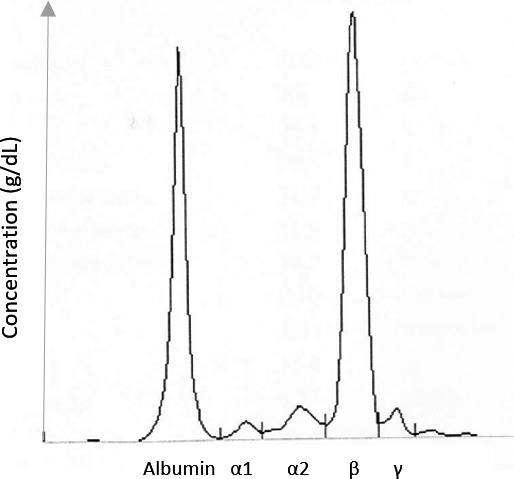
Protein electrophoresis at admission with monoclonal band in the beta region (4.3 g/dL).

Protein electrophoresis detected a monoclonal band in the beta region (4.3 g/dL) – (Fig. 1). Further study revealed IgG kappa monoclonal component (Table 1). Bone marrow aspirate demonstrated an 82% infiltration by atypical plasma cells, and FISH test was positive for gain (1q+) – intermediate‐risk abnormality – and negative for aneuploidy (5, 9, and 15), del (17p), t (4:14), and t (11:14). Serum calcium, renal function, and skeletal X‐ray bone survey were normal. Thoraco‐abdominal‐pelvic CT scan revealed several lytic lesions in the dorsal vertebral body. Bence Jones protein test was negative. The diagnosis of MM was established (MM IgGK, ISSII, 1q+ in November 2015).

Patient was classified as stage II (serum albumin 3, 6 g/dL, beta‐2 microglobulin 4, 10 mg/L, with high‐risk cytogenetic ‐1q+), according to the International Staging System (ISS) 1, 6.

Patient was referred to hematology, where induction into a chemotherapy (CT) regimen (bortezomib, cyclophosphamide, and dexamethasone = CyBorD) was proposed followed by autologous stem cell transplantation and zoledronic acid to prevent or reduce the number of skeletal lesions.

After four cycles of CyBorD, the monoclonal component was gradually decreased with treatment; aPTT was reduced/normalized; bleeding events were stopped; and anemia was resolved (Table 1). Aggregation dysfunction and LA persisted as abnormal, and the coagulation factors decreased before CT and became normal (FVIII, IX, XI, XII). Protein electrophoresis presented a monoclonal band in the beta region (0.4 g/dL), and bone marrow aspirate demonstrated a 4% infiltration by atypical plasma cells.

At that time, the patient underwent peripheral blood stem cell (PBSC) collection by apheresis technique for autologous transplantation. No bleeding, embolic episodes, or other significant adverse events occurred during the PBSC collections and the autologous transplant.

## Discussion

The major causes of acquired coagulation disorders are vitamin K deficiency, liver disease, disseminated intravascular coagulation, and the development of circulating anticoagulants 7. The first three etiologies could be excluded by a normal TP ratio, normal liver function and a normal platelets number, D‐dimers, and fibrinogen. Blood tests confirmed the presence of nonspecific coagulation inhibitors.

Bleeding events in patients with plasma cell dyscrasia not related to thrombocytopenia are uncommon 5. The case we describe showed a bleeding episode and abnormal coagulation tests due to the presence of paraproteinemia.

The association between plasma levels of paraprotein and prolonged clotting factors, particularly aPTT, has been described previously 5. Abnormal aPTT was related to the presence of LA and abnormal clotting factors (VIII, IX, and XI) resulting in a paraneoplastic syndrome related to MM, as these results normalized after chemotherapy and transplant 8, 9.

The final diagnosis of the gross hematuria was delayed, as other causes were considered, such as urinary infection and microlithiasis.

The most common presenting symptoms of MM are fatigue and bone pain. Anemia is common and contributes to fatigue. Osteolytic skeletal lesions are detected in approximately 80% of patients. Other common findings are hypercalcemia and elevated serum creatinine level. Some patients present recurrent infections as a sign of the disease 1.

Similar to this case report, there are other atypical presentations in patients with MM described in the literature, such as myelomatous pleural effusion, extramedullary plasmacytoma, headache, parasellar syndrome, and cranial nerve palsies 10, 11, 12.

It is important to be aware of the fact that the monoclonal band is usually in the gamma region, but it can also present in the beta‐ or alpha‐2 regions 2.

In the presented case, Bence Jones proteins were negative. Serum free light chains are only present in urine if the capacity for absorption in the proximal tubules is exceeded by plasma cells production. This means, to be positive, production must be significantly raised and/or the renal function must be significantly impaired 13.

Plain radiography only demonstrates lytic bone disease when 30% or more of trabecular bone has been lost 14. In this patient, although plain radiography of the entire skeleton did not show lytic lesions, the CT scan, which was used for diagnosis of occult neoplasia, was important for the decision to treat with zoledronic acid.

In 2014, the International Myeloma Working Group updated the diagnostic criteria for MM by adding three highly specific biomarkers to the existing classic markers of end‐organ damage (hypercalcemia, renal insufficiency, anemia, or bone lesion), which are clonal bone marrow plasma cells ≥60%, serum free light chain ratio > 100, and >1 focal bone lesion on magnetic resonance imaging 1, 15, 16. The diagnosis of multiple myeloma requires at least 10% of plasma cells on bone marrow examination or a biopsy proving plasmacytoma plus or at least one specific biomarker or classic marker of end‐organ damage 1. This update allows early diagnosis and initiation of therapy before end‐organ damage 1.

Prognosis in multiple myeloma depends on several factors such as host factors, tumor stage, cytogenetic abnormalities, and response to therapy. Median survival is 5 to 7 years. The revised International Staging System combines tumor stage and disease biology (cytogenetic abnormalities or elevated lactate dehydrogenase level) 1. Our patient was classified as stage II in this prognostic index, which is the most frequent stage and confers a rate of 62% for 5‐year survival 1.

In conclusion, the uncommon case presented here highlights the need to be alert to the atypical manifestation of paraproteinemia and to consider different etiologies for hematuria after excluding the most common ones, particularly when it is associated with other abnormal blood tests, such as coagulation parameters, especially in older patients.

## Authorship

MA: responsible for study design, data collection, data interpretation, manuscript preparation, and literature search. RM: collaborated in data collection, data interpretation and manuscript preparation, and review. FR and AR: helped in data interpretation, literature search, manuscript preparation, and review. TF: responsible for manuscript preparation and review.

## Conflict Of Interest

The authors declare that they have no conflict of interest and did not receive any financial support.
